# Hypocellular scar formation or aberrant fibrosis induced by an intrastromal corneal ring: a case report

**DOI:** 10.1186/1752-1947-5-398

**Published:** 2011-08-19

**Authors:** Xiaoguang Cao, Roxana Ursea, Defen Shen, Hema L Ramkumar, Chi-Chao Chan

**Affiliations:** 1 Immunopathology Section, Laboratory of Immunology, National Eye Institute, National Institutes of Health, Bethesda, MD, USA; 2 Department of Ophthalmology, People's Hospital, Peking University, Beijing, China; 3 Department of Ophthalmology & Visual Science, University of Arizona, AZ, USA; 4 Howard Hughes Medical Institute, Chevy Chase, MD, USA

## Abstract

**Introduction:**

Intrastromal corneal rings or segments are approved for the treatment of myopia and astigmatism associated with keratoconus. We describe a clinicopathological case of intrastromal corneal rings. For the first time, the molecular pathological findings of intrastromal corneal rings in the cornea are illustrated.

**Case presentation:**

A 47-year-old African-American man with a history of keratoconus and failure in using a Rigid Gas Permeable contact lens received an intrastromal corneal ring implant in his left eye. Due to complications, penetrating keratoplasty was performed. The intrastromal corneal ring channels were surrounded by a dense acellular (channel haze) and/or hypocellular (acidophilic densification) collagen scar and slightly edematous keratocytes. Mild macrophage infiltration was found near the inner aspect of the intrastromal corneal rings. Molecular analyses of the microdissected cells surrounding the intrastromal corneal ring channels and central corneal stroma revealed 10 times lower relative expression of *IP-10/CXCL10 *mRNA and two times higher *CCL5 *mRNA in the cells surrounding the intrastromal corneal ring, as compared to the central corneal stroma. IP-10/CXCL10 is a fibrotic and angiostatic chemokine produced by macrophages, endothelial cells and fibroblasts.

**Conclusion:**

An intrastromal corneal ring implant can induce hypocellular scar formation and mild inflammation, which may result from aberrant release of fibrosis-related chemokines.

## Introduction

Intrastromal corneal ring segments (Intacs, KeraVision, Inc., Fremont, CA, USA), which have been approved for the treatment of myopia and astigmatism associated with keratoconus, are two tiny half rings made of polymethyl methacrylate [1]. Compared with penetrating keratoplasty or laser surgery, Intacs has the advantage of reshaping the abnormal cornea without removing tissue or injuring the central cornea, which is most critical for clear vision. Studies have shown that Intacs could be easily removed, with reversal of visual, refractive, and topographic changes.

After implantation of Intacs, an extracellular intrastromal material may be found in the lamellar channel surrounding the segments [2]. This material might spontaneously dissolve after removal of the Intacs segments. Histopathology of corneas implanted with Intacs has been reported, and this material has been referred to as acidophilic densification and/or channel haze [3,4]. We describe, for the first time, the molecular histopathological findings of a corneal button with Intacs rings. The study was approved by the US National Eye Institute Institutional Review Board for human subjects, and the patient signed an informed consent.

## Case presentation

**Figure 1:**
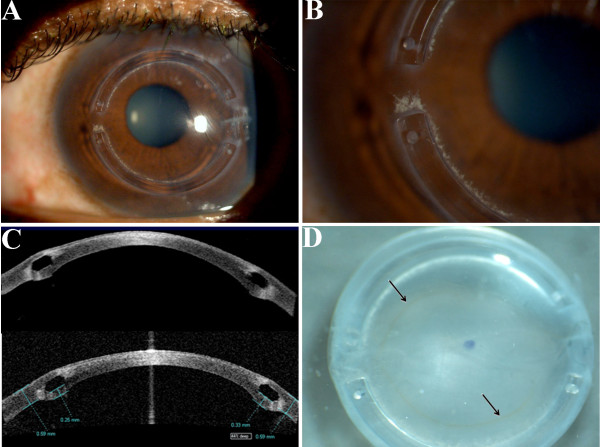
**Clinical and gross photos of Intacs (intrastromal corneal rings)**. A, The slit lamp examination of the Intacs; B, The slit lamp examination shows whitish deposit around the Intacs; C, Visante Images of the Intacs; D, Gross photo of corneal button shows the Intacs segments and Fleischer ring (arrow).

A 47-year-old African-American man with bilateral keratoconus, myopia and high astigmatism had no family history, allergies, or eye rubbing. In 2008, his best-corrected visual acuity was 20/25 for the right eye and 20/80-2 for the left eye. He presented with a clear cornea, significant cone with inferior steepening confirmed on topography, and positive Munson signs. He was diagnosed with keratoconus in 2009, and both spectacles and contact lenses correction failed to improve his vision. The patient's left eye had significant steepening and irregular astigmatism with 9 diopters of cylinder at 65 degrees. Intacs were implanted in both eyes in accordance with the patient's wishes in 2009. Post-operatively, anterior segment optical coherence tomography (AS-OCT, Visante, Carl Zeiss Meditec, Jena, Germany) imaging revealed both Intacs segments placed deep in the stroma in good position with temporal sutures in place in both eyes (Figure 1A-C). Rigid gas-permeable lenses fitted well over the cornea in the right eye, but poorly in the left eye. Ten months after the placement of the Intacs, there were multiple, whitish, small crystalline deposits around the edges and the border of the superior Intacs segment nasally in the left eye. Due to persistent poor vision, the patient elected to undergo penetrating keratoplasty on the left eye, which was successfully performed.

**Figure 2:**
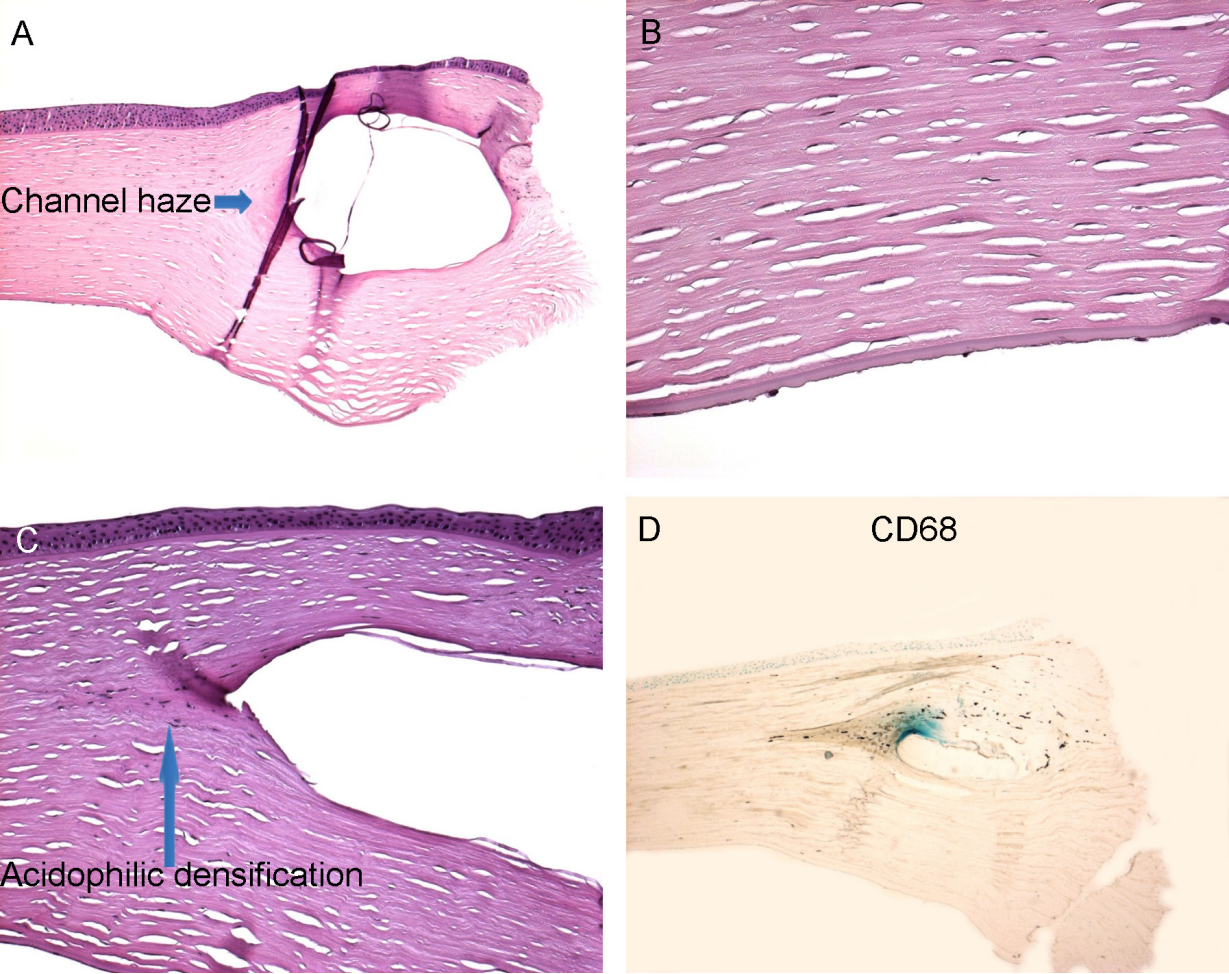
**Histopathology of Intacs (intrastromal corneal rings)**. A, Shows a space containing residue plastic (Intacs) material which is surrounded by a dense acellular and/or hypocellular collagen scar. The channel haze is observed at the inner edge of the Intacs. B, The severely attenuated corneal endothelial cells. C, Shows the acidophilic densification and mild inflammatory cell infiltration at the inner edge of the Intacs. D, Immunochemistry of CD68 shows macrophage (CD68+) infiltration at the inner edge of Intacs. (A-C, H+E; original magnification, A × 100, B and C × 200, D × 100).

The transparent corneal button with a visible Fleischer ring, measured 8.5 mm in diameter and contained two semi-circular transparent rings (Intacs) in the peripheral stroma (Figure 1D and Figure 2A). Microscopy revealed that the central stroma was thin, and the endothelia were severely attenuated and decreased (Figure 2B), which is consistent with advanced keratoconus. The epithelial cells were flattened centrally and were irregular or missing peripherally. A 1.0 mm × 0.5 mm space containing residual plastic (Intacs) material was surrounded by a dense acellular (channel haze) and hypocellular (acidophilic densification) collagen scar, particularly in the inner border (Figure 2C). The keratocytes near the Intacs were slightly edematous. There was mild macrophage (CD68+ cells) infiltration, mainly at the inner aspect of the Intacs (Figure 2D). Terminal deoxynucleotidyl transferase dUTP nick end labeling (TUNEL) staining revealed occasional positive cells around the Intacs. The cells surrounding the Intacs and central corneal stroma were separately microdissected and subjected for quantitative RT-PCR as published previously [5]. Molecular analysis normalized with the relative expression to Universal Human Reference RNA calculated 10.22 times lower *Interferon inducible protein-10/chemokine (C-X-C motif) ligand 10 (IP-10/CXCL10) *mRNA and 1.78 times higher *CCL5 *mRNA in the fibrous tissue surrounding the Intacs, as compared to the central corneal stroma.

## Discussion

Intacs have been used for several years and studied with focuses on clinical and refractive outcomes. Only a few histopathological reports are published [1,3], in which a dense acellular (channel haze) and hypocellular (acidophilic densification) collagen scar, and slightly edematous keratocytes are described. Our case also presents these findings, which indicate aberrant fibrosis [1,3].

IP-10/CXCL10, a CXC chemokine, which promotes chemoattraction but inhibits angiogenesis, is secreted by monocytes, endothelial cells, fibroblasts and keratinocytes. IP-10/CXCL10 regulates wound healing and inhibits fibrosis in the lung [6]. IP-10/CXCL10 also induces apoptosis in several cell types. Therefore, it is not surprising that only occasional apoptotic cells are detected in the collagen scar tissue.

In our patient, the whitish deposits and macrophages were mainly located at the inner edge of the Intacs. Thus, the Intacs can induce tissue damage and secondary inflammation, which likely represent the clinical crystalline deposits. CCL5, a CC chemokine, is also called regulated upon activation, normal T-cell expressed and secreted chemokine (RANTES). Secreted by monocytes, neutrophils, T-lymphocytes, dentritic cells and fibroblasts, CCL5 plays an active role in recruiting leukocytes and is a powerful leukocyte activator [7]. The mildly increased *CCL5/RANTES *mRNA in the tissue surrounding the Intacs, as compared to the central corneal stroma, suggests active inflammation around the Intacs, especially near the inner edge.

## Conclusion

An Intacs implant induces hypocellular scar formation or aberrant fibrosis, and mild focal inflammation, possibly through its strong suppression of *IP-10/CXCL10 *production and mild enhancement of *CCL5 *secretion.

## Abbreviations

Intacs: Intrastromal corneal ring segments.

## Consent

Written informed consent was obtained from the patient for publication of this case report and accompanying images. A copy of the written consent is available for review by the Editor-in-Chief of this journal.

## Competing interests

The authors declare that they have no competing interests.

## Authors' contributions

XC performed the pathological examination and molecular analysis, and wrote the manuscript draft. RU provided patient care and clinical information, submitted the specimen, and reviewed the manuscript. DS performed and analyzed the molecular analysis, and reviewed the manuscript. HLR analyzed and reviewed the clinical, histopathological and molecular data, and edited the manuscript. CCC designed and supervised the study, interpreted pathological and molecular findings, and critically reviewed the manuscript. All authors read and approved the final manuscript.
